# Recent progress in self-healable ion gels

**DOI:** 10.1080/14686996.2020.1777833

**Published:** 2020-06-24

**Authors:** Ryota Tamate, Masayoshi Watanabe

**Affiliations:** a Center for Green Research on Energy and Environmental Materials, National Institute for Materials Science, Tsukuba, Japan; b Institute of Advanced Sciences, Yokohama National University, Yokohama, Japan

**Keywords:** Ionic liquids, ion gels, photo-healing, thermal-healing, self-healing, gel electrolytes, wearable electronics, stretchable electronics, 101 Self-assembly / Self-organized materials, 201 Electronics / Semiconductor / TCOs, 206 Energy conversion / transport / storage / recovery

## Abstract

Ion gels, soft materials that contain ionic liquids (ILs), are promising gel electrolytes for use in electrochemical devices. Due to the recent surge in demand for flexible and wearable devices, highly durable ion gels have attracted significant amounts of attention. In this review, we address recent advances in the development of ion gels that can heal themselves when mechanically damaged. Light- and thermally induced healing of ion gels are discussed as stimuli-responsive healing strategies, after which self-healable ion gels based on supramolecular and dynamic covalent chemistry are addressed. Tough, highly stretchable, and self-healable ion gels have recently been fabricated through the judicious design of polymer nanostructures in ILs in which polymer chains and IL cations and anions interact. The applications of self-healable ion gels to electrochemical devices are also briefly discussed.

## Graphical abstracts

**Figure:**
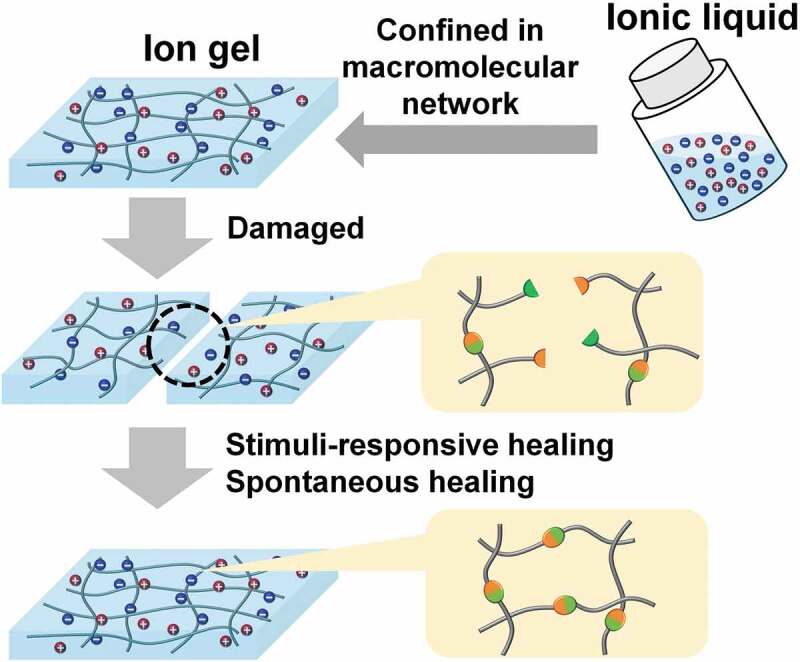

## 1. Introduction

**Figure 1.**
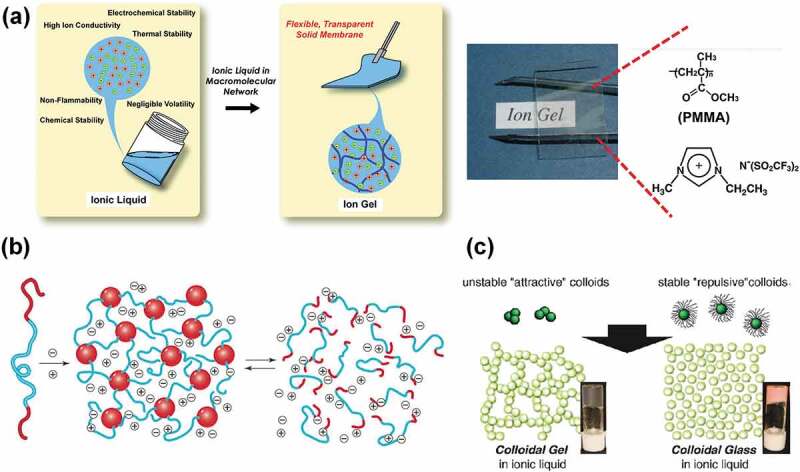
(a) Preparation of a chemically crosslinked ion gel by the in situ radical polymerization of a vinyl monomer, such as methyl methacrylate, in an IL. Reproduced with permission from ref. 7. American Chemical Society 2008. (b) Ion gel formation through the self-assembly of an ABA triblock copolymer in an IL. The physical association can be reversible by suitable choice of the A block and the IL. Reproduced with permission from ref. 19. American Association for the Advancement of Science 2008. (c) A colloidal ion gel and a glass composed of silica nanoparticles in ILs. Reproduced with permission from ref. 31. American Chemical Society 2011.

Ionic liquids (ILs) are room temperature molten salts solely composed of cations and anions. ILs possess very attractive features as electrolytes, including high ionic conductivities, nonflammability, negligible vapor pressures, and high chemical/electrochemical stabilities. Through the judicious selection of cations and anions, many classes of IL have been designed for use in a variety of electrochemical devices, such as supercapacitors, lithium secondary batteries, fuel cells, electric double layer transistors, and soft actuators [1–6]. For practical applications, however, it is desirable to endow ILs with solid-like mechanical integrity while keeping the attractive properties of ILs. Therefore, IL-based gel electrolytes, referred to as ‘ion gels’, have been intensively studied over the past few decades [7–11]. Compared to conventional gel electrolytes that contain water or organic solvents (i.e., hydrogels and organogels), ion gels satisfy the requirements of high levels of safety, durability at elevated temperatures and in the open atmosphere, and excellent electrochemical properties that are attributable to the intrinsic nature of ILs. To date, a variety of strategies have been proposed to gelate ILs. Watanabe and co-workers first prepared chemically crosslinked ion gels by the in situ radical polymerization of IL-compatible vinyl monomers with a small amount of a crosslinker in the IL (Figure 1a) [12,13]. Certain polymer/IL systems were later shown to exhibit lower critical solution temperature (LCST), as well as upper critical solution temperature (UCST) phase behavior during solubility studies involving various polymers in ILs [14–17]. These findings provide pathways for the fabrication of intelligent ion gels that undergo temperature-dependent volume phase transitions. In addition to covalent chemical crosslinking, the physical association of block copolymers can also be exploited in order to gelate ILs (Figure 1b) [9,18,19]. Lodge and co-workers demonstrated that ABA-type triblock copolymers in ILs form ion gels through self-assembly, in which the physically associated IL-phobic A blocks are bridged by IL-philic B blocks, thereby forming three-dimensional percolated networks [20–22]. One of the advantages of block-copolymer-based ion gels lies in the reversibility of the physical association, which enables the facile solution printing of ion gels onto substrates [23–26]. In addition, temperature-dependent reversible sol-gel transitions of ion gels can be realized by introducing temperature-responsive polymers into the A block [27–29]. Moreover, not only polymeric materials but also inorganic materials can be used to fabricate ion gels [30–35]. It was reported by Watanabe and co-workers that a colloidal gel and a colloidal glass were formed in ILs depending on the colloidal stability of silica nanoparticles in ILs (Figure 1c) [31,32]. Aida and co-workers demonstrated that simply grinding carbon nanotubes (CNTs) with imidazolium-based ILs resulted in the formation of gel-like pastes, referred to as ‘bucky gels’, which was attributed to the debundling of the CNTs through cation-ᴨ interactions between the CNT surfaces and the imidazolium cations [33–35].

Ion gels have been proposed for use in various electrochemical devices. The advent of the Internet of Things (IoT) era has especially seen an increase in the demand for wearable/stretchable electrochemical devices that can be operated under repeated mechanical deformation for long periods of time [36–38]. Due to their high liquid contents, gel materials generally suffer from inferior mechanical properties [39]. In this context, improving the mechanical strengths of ion gels while maintaining their superior electrochemical properties represents a great challenge for their future use in wearable and stretchable technologies. Among many strategies for enhancing the mechanical properties of a material, the ability to self-heal is among the most promising functions; self-healing can realize unprecedented durable materials by repairing damage through specific stimuli or in an autonomic fashion [40–46]. Despite their importance, ion gels with healing functions remain developed only at rudimentary levels. In this review, we provide an overview of recent advances in novel ion gels that have the ability to heal following mechanical damage. In the following section, we highlight healable ion gels that respond to specific stimuli by forming and collapsing stimuli-responsive networks. Several types of self-healable ion gel that function in the absence of external stimuli will then be addressed. Finally, possible applications of healable ion gels are briefly discussed.

## 2. Stimuli-induced healing of ion gels

### 2.1. Photo-healable ion gels

**Figure 2.**
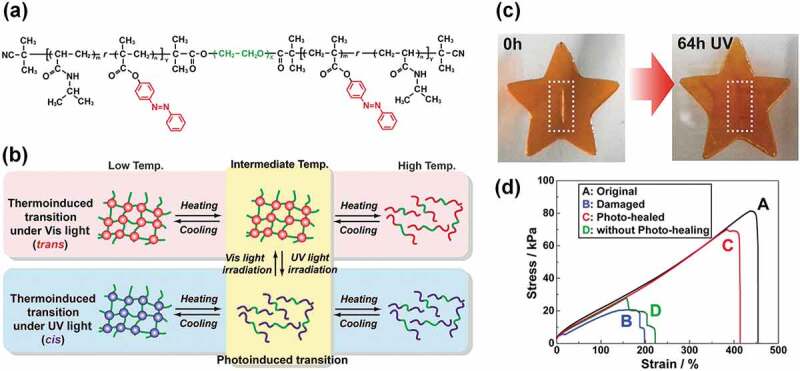
(a) Chemical structure of the photo/thermo-responsive ABA triblock copolymer. (b) Schematic illustration of the sol-gel transition of the ABA triblock copolymer in an IL when irradiated with visible or UV light. An intermediate temperature range exists where the photo-induced sol-gel transition is realized due to different gel-to-sol transition temperatures. Reproduced with permission from ref. 58. Wiley 2015. (c) Photo-healing of the damaged ion gel through the UV light-induced gel-to-sol transition process. (d) Tensile stress–strain curves for ion gels in different states: (A) original, (B) damaged, (C) photo-healed after damage, (D) simply aged after damage. The IL content in the ion gel is 80 wt%. Reproduced with permission from ref. 59. American Chemical Society 2015.

Photo-induced healing is very attractive due to the non-invasiveness and high spatiotemporal resolution of light [47–53]. Generally, photo-induced healing relies on the reversible formation and disintegration of chemical or physical crosslinking points triggered by light-induced chemical reactions or molecular conformational changes. Azobenzene, which undergoes wavelength-dependent isomerization between its *cis* and *trans* states, is among the most well-known photochromic molecules [54–57]. Photo-healable ion gels were realized by introducing an azobenzene-containing thermoresponsive polymer as one block of an ABA triblock copolymer [58–60]. Poly(*N*-isopropyl acrylamide) (PNIPAm) showed a UCST-type phase transition in several imidazolium-based ILs [14]. When 4-phenylazophenyl methacrylate (AzoMA), a methacrylate monomer containing the azobenzene chromophore, was copolymerized with NIPAm, the UCST of the P(NIPAm-*r*-AzoMA) copolymer in an IL was found to largely depend on the isomerization state of the azobenzene moiety [61]. We demonstrated that reversible sol-gel transitions can be realized using the P(NIPAm-*r*-AzoMA) copolymer as end blocks of an ABA triblock copolymer and by switching the wavelength of the light source [58]. The well-defined ABA triblock copolymer, composed of a photo- and thermo-responsive P(NIPAm-*r*-AzoMA) as the A block and an IL-philic poly(ethylene oxide) (PEO) as the B block, was synthesized by living radical polymerization (Figure 2a). The ABA triblock copolymer solution in 1-butyl-3-methylimidazolium hexafluorophosphate, an aprotic IL, exhibited a gel-to-sol transition as the temperature was increased due to the disassembly of the physically associated end block through UCST-type phase behavior. The sol-gel transition temperature depends on the isomerization state of the azobenzene moiety, which is the key factor for realizing photo-healing. As a result, an intermediate temperature is observed, where the triblock copolymer solution exists in the sol-state when irradiated with UV light, whereas it forms a gel under visible light (Figure 2b). Hence, this unique feature was used to achieve the photo-healing function [59]. When the triblock copolymer ion gel suffers mechanical damage in the intermediate temperature range, exposure of the damaged area to UV light induces the gel-to-sol transition, which leads to fluidization and filling of the damaged region (Figure 2c). Subsequently, irradiation with visible light recovers the gel state in which the previously damaged part is healed. The photo-healing efficiency of the ion gel was quantified by tensile testing before and after ion gel sheets were photo-healed. Although the present system requires a long photo-healing time (~64 h), a high healing efficiency of 80% was achieved by sequential irradiation with UV and visible light (Figure 2d). A recent study clarified that the glassy and rubbery states of the P(NIPAm-*r*-AzoMA) block domains are responsible for the gel and sol states of the block copolymer system [62]. The strategy of using the photo-induced sol-gel transition was expanded to an azobenzene-containing ABC-type triblock copolymer ion gel, in which light switching induced the structural transition between jammed micelles and micellar networks [63]. In addition, it is not necessary for the azobenzene moiety to be attached to the polymer backbone; ion gels based on the thermoresponsive ABA triblock copolymer and an azobenzene-containing IL were demonstrated to undergo photo-induced reversible sol-gel transitions [64,65]. It is very interesting to note that the sol-gel transition temperature can be altered by the addition of only a few mol% of the azobenzene containing IL [64,65]. These results highlight the universality of the strategy that uses photochromic isomerization as the molecular switch that triggers the photo-healing behavior of an ion gel. It was reported that enthalpy and entropy of mixing (Δ*H*_mix_ and Δ*S*_mix_) for thermoresponsive polymer/IL systems were much smaller than those for polymer/water systems [7,17], implying that LCST and UCST for polymer/IL systems are determined by a subtle balance of Δ*H*_mix_ and Δ*S*_mix_. This would be the reason that the large difference in sol-gel temperature of ion gels was induced by small changes in the chemical structures of the polymer and IL.

**Figure 3.**
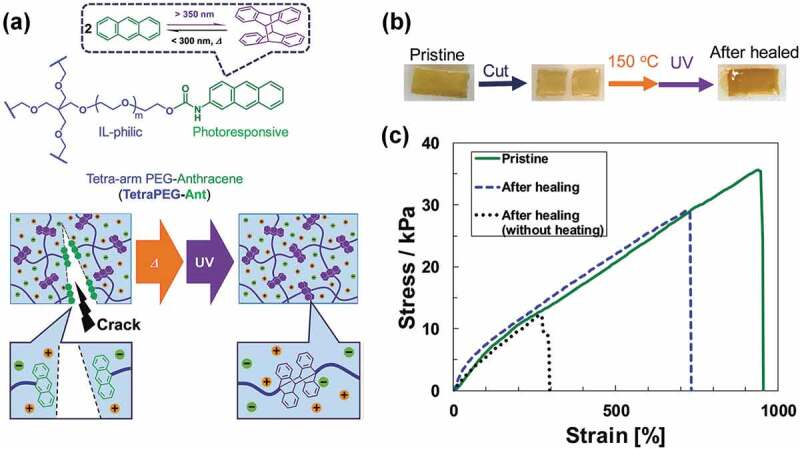
(a) Chemical structure of the anthracene-modified 4-arm PEG (tetraPEG-Ant) and schematic illustration of the photo-healable ion gel based on the reversible photodimerization reaction of anthracene in an IL. (b) Photographic images of the photo-healing process of the tetraPEG-Ant ion gel: Pristine (left), cut (center), and photo-healed (right) ion gels. (c) Tensile testing of the pristine ion gel (green solid line), the healed ion gel following heating and UV irradiation (blue dashed line), and the healed ion gel that was only UV irradiated (black dotted line). The IL content in the ion gel is 90 wt%. Reproduced with permission from ref. 69. Royal Society of Chemistry 2018.

In addition to photo-isomerization, ion gel photo-healing can also be realized by exploiting photo-dimerization reactions. Chromophores, such as cinnamic acid, coumarin, and anthracene, that form dimers upon irradiation with light through a [2ᴨ-2ᴨ] or [4ᴨ-4ᴨ] cycloaddition mechanism, have been introduced into polymer architectures to fabricate photo-responsive polymeric materials [53,66–68]. Importantly, these photodimerization reactions are reversible by simply changing the wavelength of light. In particular, the anthracene moiety that undergoes the [4ᴨ-4ᴨ] cycloaddition reaction reverts by heating as well as irradiation with light. Recently, we developed photo-healable ion gels that exploit the photodimerization of the anthracene moiety as a reversible chemical crosslinking point (Figure 3a) [69]. Tetra-arm poly(ethylene glycol) (tetraPEG) terminally modified with anthracene moieties was synthesized and combined with 1-ethyl-3-methylimidazolium bis(trifluoromethanesulfonyl)amide ([C_2_mim][NTf_2_]). Ion gels were formed by UV irradiation at a wavelength near 350 nm when anthracene-modified tetraPEG (tetraPEG-Ant) was dissolved in the IL at a polymer concentration higher than the overlapping concentration, as the generated anthracene photo-dimer at the polymer terminal behaves as a chemical crosslinking point. Since the chemical crosslink formed by the anthracene dimer involves a covalent bond that is weak compared to the carbon-carbon covalent bonds in the polymer main chain, the anthracene photodimerization site in the gel network is anticipated to preferentially cleave when the ion gel is mechanically damaged. By exploiting the dynamic nature of the anthracene photodimer, a cut surface of the ion gel was recovered by thermal annealing and subsequent irradiation with UV light (Figure 3b). Thermal annealing prior to irradiation with UV-light was necessary to promote photo-healing behavior, probably because the higher concentration of monomeric anthracene generated by thermal treatment enhances the bimolecular reaction of the terminal anthracene moiety at the cut surface. Tensile testing of the tetraPEG-Ant ion gels before and after photo-healing revealed that the ion gel was highly stretchable even after healing, with an elongation at break of about 700% and a healing efficiency of 66% (Figure 3c). This research demonstrated that the photodimerization-based healing of ion gels is possible; however, the mechanical properties of these ion gels deteriorate by repeated photo-healing because of insufficient reversibility of the photodimerization reaction. Therefore, increasing the reversibility of the photo-dimerization reaction in the IL is critical in order to further improve photo-healing efficiency.

### 2.2. Thermally healable ion gels

**Figure 4.**
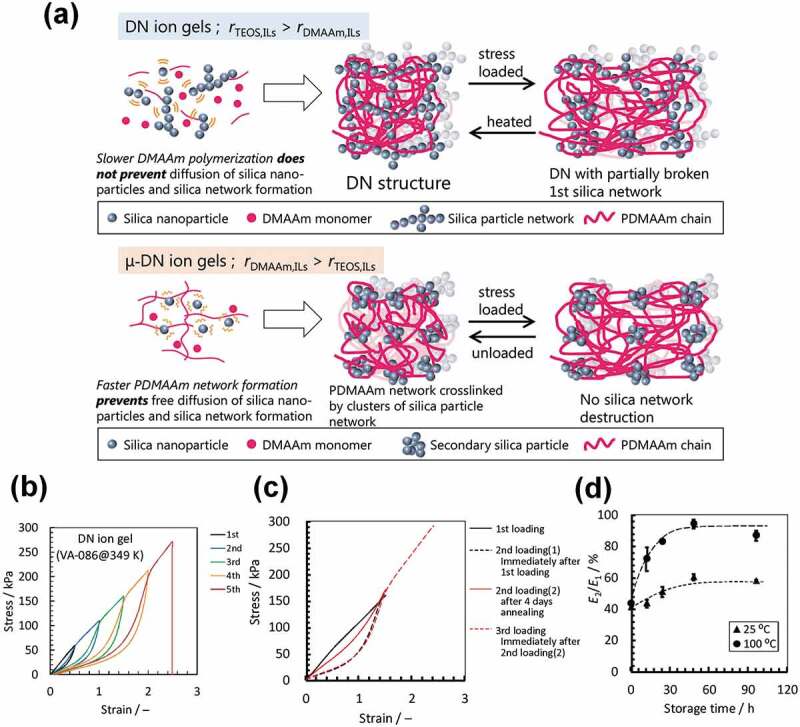
(a) Schematic illustration of the fabrication processes for a DN ion gel and a μ-DN ion gel formed by inorganic/organic networks. (b) Cyclic tensile testing for the DN ion gel. Clear mechanical hysteresis is observed after re-loading. (c) Recovery of the mechanical hysteresis of the DN ion gel by thermal annealing at 373 K for 4 d. (d) Recovery efficiencies of the Young’s moduli of DN ion gels stored at 25 and 100°C. The IL content in the ion gel is 80 wt%. Reproduced with permission from ref. 70. Wiley 2017.

Kamio and co-workers developed highly tough and thermally healable double network (DN) ion gels consisting of organic and inorganic networks [70–72]. The toughening mechanism in the DN concept relies on the introduction of two dissimilar networks; i.e., a rigid and brittle network and a soft and ductile network [73,74]. A brittle and rigid inorganic network was formed in the DN ion gel through physical bonding (aggregation) between silica nanoparticles synthesized by the polycondensation of tetraethoxysilane. On the other hand, the soft and ductile network was chemically crosslinked poly(*N,N*-dimethyl acrylamide) (PDMAAm) synthesized by free radical polymerization. Two types of ion gel were prepared by controlling the reaction kinetics for the formation of each network in the IL (Figure 4a). Both networks percolated through the ion gel (DN ion gel) when the silica nanoparticle network formed faster than the PDMAAm network. In contrast, when the PDMAAm network was formed first, the formation of the percolated network of silica nanoparticles was suppressed by the as-formed polymer network, leading to spatially dispersed silica clusters in the PDMAAm network (μ-DN ion gel). Although μ-DN ion gels also exhibit better tensile properties than ion gels formed by single PDMAAm networks, mechanical hysteresis and softening behavior during cyclic tensile testing, both of which are typical characteristics of DN gels, were not observed. On the other hand, DN ion gels showed superior tensile properties to μ-DN ion gels, with mechanical hysteresis observed (Figure 4b), which is attributable to energy dissipation through the destruction of the physically bonded silica network under extension, and indicates that the silica network functions as a sacrificial bond. Remarkably, in addition to their excellent mechanical properties, the organic/inorganic DN ion gels exhibited healing behavior for mechanical hysteresis as well as Young’s modulus by thermal annealing. Hysteresis does not recover in conventional DN gels formed by two asymmetric chemically crosslinked polymer networks due to permanent damage caused to the first brittle and rigid network. As the brittle and rigid network in an organic/inorganic ion gel is constructed through physical interactions between silica nanoparticles, the network can reform by the rearrangement of fragmented small silica clusters. In Figure 4c, softening behavior was observed for the stress-strain curves of the second and third loading immediately after the previous loading. On the other hand, the softening was recovered when the second loading was conducted after 4 days annealing at 373 K. At room temperature, the healing efficiency, as determined by the Young’s modulus and toughness, is moderate because of the restricted motion of the silica clusters. However, excellent recovery was observed by elevating the temperature (Figure 4d). Physical interactions between nanoparticles, such as hydrogen bonding and van der Waals forces, are weakened at high temperature, which accelerates network rearrangement.

**Figure 5.**
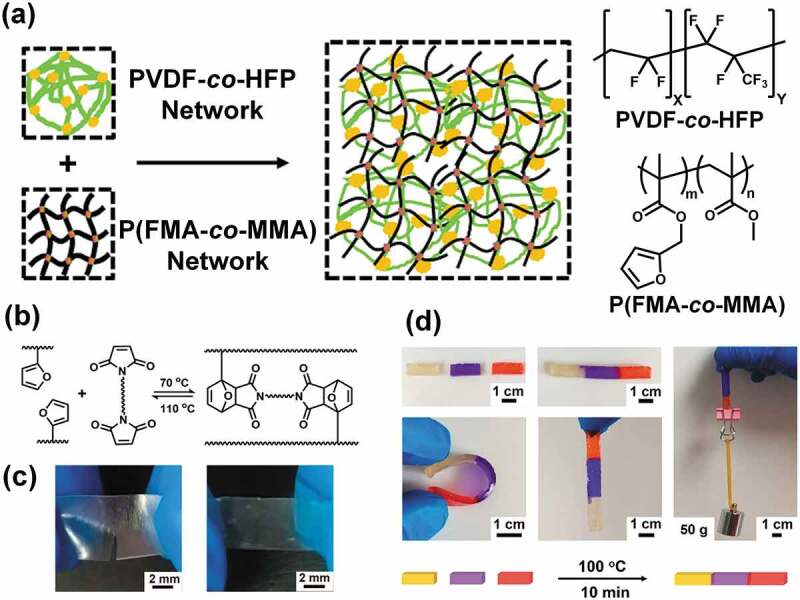
(a) Schematic illustration of a DN ion gel composed of a physically crosslinked PVDF-*co*-HFP network and a chemically crosslinked P(MMA-*co*-FMA) network formed by a thermally reversible Diels-Alder reaction. (b) Reaction formula for the thermally reversible furan–maleimide Diels-Alder reaction. (c) Fast thermal-healing behavior of a DN ion gel film at 100°C for 20 s. (d) Thermal healing of the bulk ion gel at 100°C for 10 min. The healed ion gel is freely bendable and can bear a 50 g weight. The IL content in the ion gel is 80 wt%. Reproduced with permission from ref. 75. American Chemical Society 2018.

More recently, Fan and co-workers use a thermally reversible Diels-Alder (DA) reaction to realize thermally healable ion gels [75]; they also designed ion gels with the DN structure, in which physically crosslinked poly(vinylidene fluoride-*co*-hexafluoropropylene) (PVDF-*co*-HFP) provided a soft and ductile network, and a chemically crosslinked poly(furfuryl methacrylate-*co*-methyl methacrylate) (P(FMA-*co*-MMA)) functioned as a rigid and brittle network (Figure 5a). The ion gel was synthesized in a one-pot process; PVDF-*co*-HFP, P(FMA-*co*-MMA), *N,N’*-(4,4ʹ-diphenylmethane)bismaleimide (BMI), and [C_2_mim][NTf_2_] were dissolved in acetone. The first physically crosslinked PVDF-*co*-HFP network was spontaneously formed by crystallization of the PVDF upon evaporation of the acetone, and subsequent heating at 70°C caused P(FMA-*co*-MMA) and BMI to chemically crosslink through furan-maleimide DA reactions. The DN ion gel with the optimum composition was much more stretchable and tough compared to single PVDF-*co*-HFP or P(FMA-*co*-MMA) ion gels. More importantly, the furan-maleimide DA reaction is thermally reversible, with the retro-DA reaction proceeding at around 110°C (Figure 5b). Therefore, chemical crosslinking is expected to become dynamic at an appropriately high temperature, where both de-crosslinking and re-crosslinking reactions occur. As a result, fast thermal healing of the partially cut ion gel film was achieved by heating at 100°C for only 20 s (Figure 5c). Even cut surfaces of bulk ion gel samples were quickly merged within 10 min by thermal annealing at 100°C, and the healed ion gels were freely bendable and weight-bearing (Figure 5d). It should be noted that it is difficult to thermally heal hydrogels and organogels due to the volatile nature of both water and organic solvents, and because most organic solvents are flammable. Therefore, it can be said that the ion gel thermal-healing strategy provides a very promising method that exploits the unique characteristics of ILs.

## 3. Self-healable ion gels

The use of reversible non-covalent or dynamic covalent interactions in self-healing materials has received significant attention [76–85]. Although still in its early stages, studies into self-healing ion gels based on supramolecular and dynamic covalent chemistries have gradually increased in number in recent years [86–90]. For example, self-healing ion gels that exploit hydrogen bonding between hydrophilic ILs and biopolymers, such as guar gum [86] and functionalized agarose [87], have been reported. The formation of dynamic covalent bonds through Schiff-base reactions in ILs was used to form dendrimer-based self-healing ion gels [88]. Judicious combinations of low-molecular-weight gelators and ILs were also shown to realize self-healable ion gels through hydrogen bonding, π − π stacking, and interactions between alkyl chains [89].

**Figure 6.**
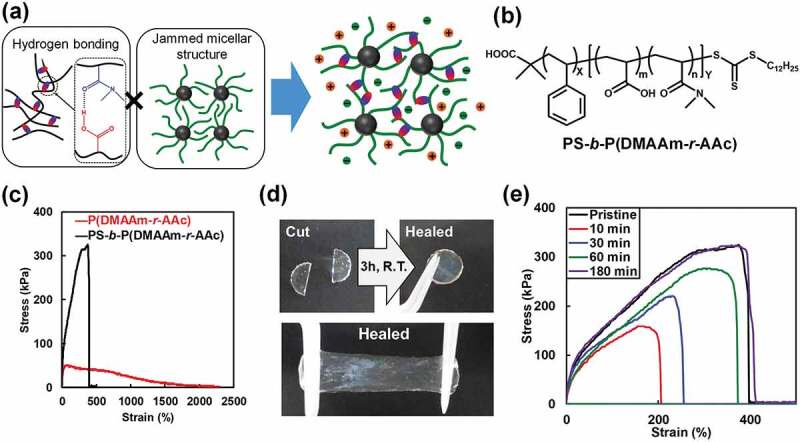
Conceptual illustration of a self-healing micellar ion gel formed through multiple hydrogen bonds and micelle jamming. (b) Chemical structure of the PS-*b*-P(DMAAm-*r*-AAc) diblock copolymer. (c) Comparing the stress–strain curves for the P(DMAAm-*r*-AAc) ion gel (red) and PS-*b*-P(DMAAm-*r*-AAc) ion gel (black). (d) Photographic images showing the self-healing behavior of the micellar ion gel at room temperature for 3 h. (e) Tensile stress–strain curves for the pristine ion gel and ion gels healed at room temperature for various times. The IL content in the ion gel is 70 wt%. Reproduced with permission from ref. 91. Wiley 2018.

Nevertheless, achieving ion gels that rapidly self-heal and are highly mechanically robust remains a challenging task. Recently, we developed tough, self-standing, and self-healable ion gels based on synergism between multiple hydrogen bonds and jammed micellar structures (Figure 6a) [91]. The micellar ion gel was composed of a diblock copolymer and [C_2_mim][NTf_2_], a conventional IL, and the diblock copolymer was composed of a polystyrene (PS) block that is incompatible with the IL, and a poly(DMAAm-*r*-acrylic acid) (P(DMAAm-*r*-AAc)) block that exhibits hydrogen bonding properties (Figure 6b). The DMAAm and AAc units in the P(DMAAm-*r*-AAc) block act as hydrogen bonding acceptors and donors, respectively, and the resulting hydrogen bonds are stronger than those to ILs consisting of low Lewis acidic and basic cations and anions, respectively. Thus, when the PS-*b*-P(DMAAm-*r*-AAc) diblock copolymer and [C_2_mim][NTf_2_] were combined, the IL-insoluble PS block aggregated to form a micelle core, whereas the hydrogen-bonding IL-compatible P(DMAAm-*r*-AAc) block acted as a micelle corona. Consequently, a percolated micellar network was formed in the IL by jammed micelles that were multiply hydrogen bonded to each other. Remarkably, the introduction of the jammed micellar structure resulted in greatly enhanced self-standing stability and mechanical strength compared to those of the ion gel formed only through the hydrogen-bonding P(DMAAm-*r*-AAc) copolymer (Figure 6c). As the hydrogen bonding interactions are transient physical crosslinks, terminal flow behavior was observed on the long timescale, and necking deformation was observed under extension, leading to poor self-standing stability and low mechanical strength for the P(DMAAm-*r*-AAc) ion gel. On the other hand, the jammed micelle structure and the multiple hydrogen bonds between micelles in the PS-*b*-P(DMAAm-*r*-AAc) ion gel suppressed the macroscopic motions of micelles and polymer-chain sliding under elongation. Therefore, the micellar ion gel exhibited significantly suppressed terminal flow behavior, a highly enhanced self-standing ability, and mechanical toughness. Furthermore, the micellar ion gel showed fast self-healing behavior at room temperature without any external stimulus. Spontaneous healing was observed within a few hours at room temperature when an ion gel that had been cut into two pieces was reattached (Figure 6d). Tensile testing of the pristine sample and ion gel samples healed for various times revealed that fracture stress and strain increasingly recover with increasing healing time, with the stress–strain curve after 3 h almost identical to that of the pristine ion gel (Figure 6e). Since hydrogen bonding between micelles is weaker than covalent bonding in the polymer main chain and the glassy PS micelle cores, hydrogen bonding is considered to be preferentially cleaved when mechanically damaged. Therefore, the hydrogen bonds between the block copolymer micelles are assumed to reform at the cut surface, resulting in fast self-healing behavior at room temperature. In addition to their mechanical properties, cyclic voltammetry of the pristine and healed ion gel sheets revealed that electrochemical properties also recover. These results highlight that synergism between reversible hydrogen bonding and the block copolymer nanostructure contribute to the fabrication of self-healing ion gels with favorable mechanical and electrochemical properties. The physical properties of the micellar ion gel were also found to be greatly affected by subtle differences in the structures of the IL cations and anions [92], which implies that controlling competitive hydrogen bonding among the cations, anions, and polymer chains is the key to achieving desirable physical properties.

**Figure 7.**
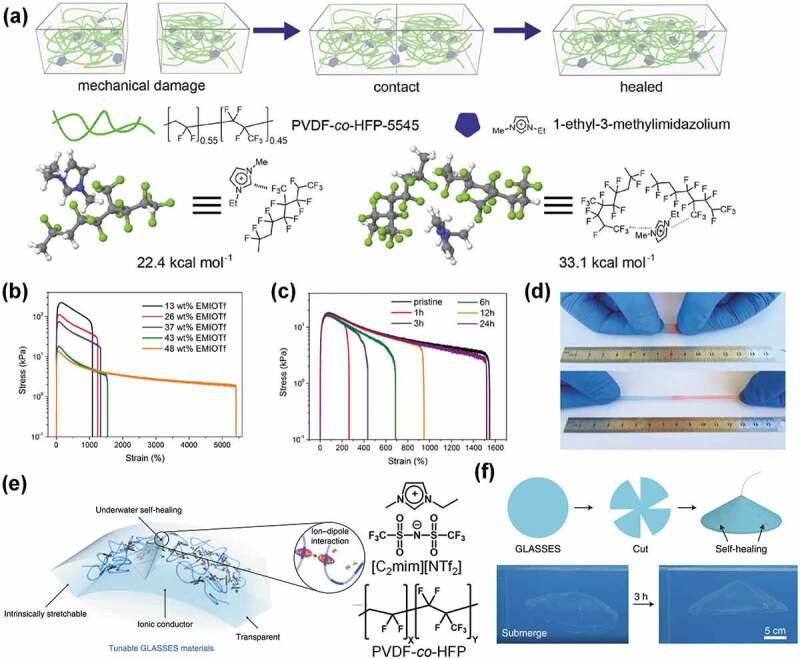
(a) Schematic image depicting the self-healing behavior of the PVDF-*co*-HFP-5545 ion gel through ion-dipole interactions with imidazolium cations. (b) Tensile stress–strain curves for ion gels with various IL contents. (c) Self-healing behavior of the ion gel at room temperature for various healing times evaluated by tensile testing. (d) Photographic images of the healed ion gel before and during stretching. The IL content in the ion gel is 43 wt% in (c) and (d). Reproduced with permission from ref. 93. Wiley 2017. (e) Conceptual illustration of the self-healable and water-resistant ion gel formed by PVDF-*co*-HFP-5545 and [C_2_mim][NTf_2_]. (f) Photographic images of the water-submerged ion gel after healing; the submerged ion gel maintained its shape and transparency for 3 h. Reproduced with permission from ref. 96. Nature Publishing Group 2019.

Keplinger, Wang, and co-workers exploited ion-dipole interactions between ILs and PVDF-*co*-HFP as the driving force to realize highly stretchable, transparent, and self-healable ion gels (Figure 7a) [93]. Ion gels composed of PVDF-*co*-HFP and ILs had already been reported [94,95], where PVDF-*co*-HFP with a high VDF content was combined with a high IL load. On the other hand, the study of Keplinger et al. used PVDF-*co*-HFP with a high (45 mol%) HFP content (PVDF-*co*-HFP-5545), which was poorly crystalline and exhibited a high dipole moment, and combined with relatively small amounts of ILs (< 50 wt%). The ion gel composed of PVDF-*co*-HFP-5545 and 1-ethyl-3-methylimidazolium trifluoromethanesulfonate ([C_2_mim][TfO]) was highly transparent and highly stretchable to over 1000% (Figure 7b). Moreover, the PVDF-*co*-HFP-5545 ion gel exhibited self-healing behavior at room temperature (Figure 7c,d). Density functional theory calculations and Fourier-transform infrared spectroscopy revealed the existence of ion-dipole interaction between imidazolium cations and CF_3_ units, which presumably is the reason for the self-healing ability of the ion gel. This hypothesis was reinforced by the observation that the ion gel prepared with PVDF-*co*-HFP having 90 mol% VDF content was very rigid and did not show any self-healing ability. The PVDF-*co*-HFP-5545 ion gel was used as a self-healable ionic conductor in dielectric elastomer actuators. When a non-self-healable ionic conductor was used as an actuator, mechanical damage to the ionic conductor resulted in poor actuation performance and delamination. Meanwhile, mechanical damage to the self-healable PVDF-*co*-HFP-5545 ion gel conductor was healed after 24 h, and the healed actuator showed comparable actuation performance to that of the pristine actuator. More recently, a water-resistant and self-healable PVDF-*co*-HFP-5545 ion gel was reported; this gel was prepared by replacing the hydrophilic [C_2_mim][TfO] with the hydrophobic [C_2_mim][NTf_2_] (Figure 7e) [96]. Due to the hydrophobic nature of both [C_2_mim][NTf_2_] and PVDF-*co*-HFP, the resultant ion gel maintained its gel integrity even in water (Figure 7f). Self-healing testing revealed a recovered ion gel toughness of 43.9% after 24 h at room temperature, and 99.1% after 24 h at 50°C. Interestingly, the ion gel was still able to self-heal in water; its toughness recovered about 22.6% of its initial value after 3 h in deionized water. Water-resilient self-healing ion gels are promising for use as electronic skin under aquatic conditions. Another example of a self-healing ion gel that uses ion-dipole interactions has also been reported; this ion gel was formed from [C_2_mim][NTf_2_] and polymer blends of PVDF-*co*-HFP and PDMAAm [97]. This PVDF-*co*-HFP had a high VDF content of 94 mol%; however, blending with PDMAAm was suggested to enhance polymer-polymer and polymer-IL ion-dipole interactions, resulting in self-healing behavior. As an alternative strategy, the introduction of reversible metal coordination bonds between Fe_3_O_4_ nanoparticles and polymer chains in a loosely crosslinked polymer network resulted in highly stretchable and self-healing ion gels [98].

## 4. Applications of self-healable ion gels

Several proof-of-concept applications based on the self-healing ion gels discussed so far have been demonstrated. As stretchable and durable ion-conducting soft materials, self-healing ion gels are promising for use as soft actuators that respond to voltage [93]. A stretchable strain sensor that relies on changes in the resistance of a self-healing ion gel when stretched was also constructed [96,98]. In addition, a highly transparent self-healing ion gel was also used in a capacitive touch screen [96].

**Figure 8.**
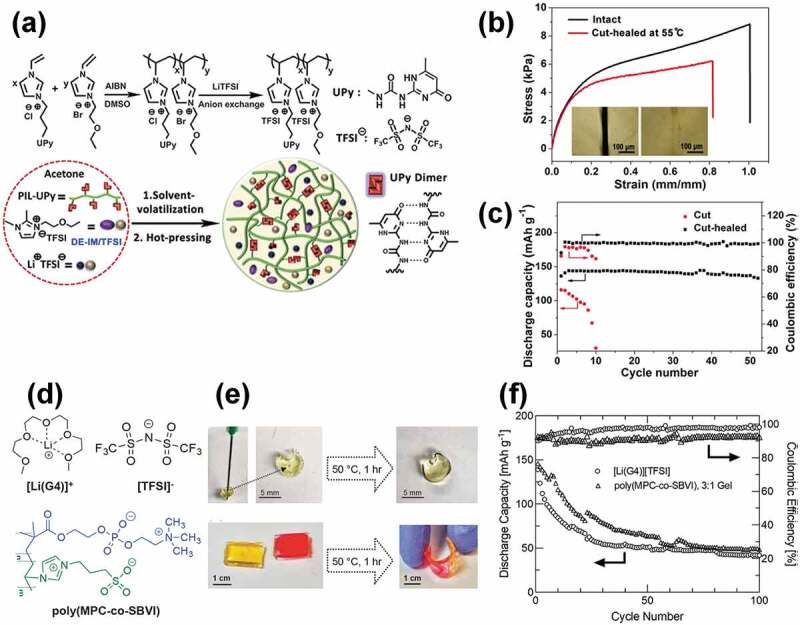
(a) Chemical structure and design concept of the healable ion gel electrolyte based on the quadruple hydrogen bonding of the UPy units and an IL. (b) Tensile stress–strain curves for the pristine ion gel and that healed at 55°C for 1 h. (c) Cycling performance of [Li | ion gel | LiFePO_4_] full cells at a 0.2 C rate. Self-healed and unhealed cut ion gels (55°C, 1 h) were used as gel electrolytes. The mass fraction ratio of the mixed IL/Li salt electrolyte and the PIL-UPy copolymer is 3.5:1 in (b) and (c). Reproduced with permission from ref. 99. American Chemical Society 2019. (d) Chemical structure of the SIL, [Li(G4)][NTf_2_], and the zwitterionic copolymer, poly(MPC-*co*-SBVI). (e) Healing behavior of punctured and cut solvate ion gels at 50°C for 1 h. (f) Cycling performance of [graphite | neat SIL or solvate ion gel | NCM(523)] full cells at a 0.5 C rate. The IL content in the ion gels is 80 mol% in (e) and (f). Reproduced with permission from ref. 100. American Chemical Society 2019.

In the IL research field, the use of ILs as electrolytes in lithium secondary batteries has been one of the most intensively studied topics due to desirable properties, such as their wide electrochemical windows and lack of volatility and flammability. In this context, healable ion gel electrolytes were recently adopted as gel electrolytes in lithium secondary batteries. Sun and co-workers reported a healable ion gel composed of imidazolium-based poly(ionic liquids) (PILs) that contain hydrogen-bonding ureido-pyrimidinone (UPy) moieties (PILs-UPy) and a mixed IL/Li salt electrolyte (Figure 8a) [99]. The quadruple hydrogen bonding functionality of the UPy moiety and electrostatic interactions between the PIL and the IL units were responsible for the good mechanical strength and self-healing ability of the ion gel. The ion gel healed at 55°C for 1 h after being cut exhibited good stress–strain properties, with 70.5% and 79.3% recoveries of tensile strength and strain at break, respectively, relative to the pristine ion gel (Figure 8b). A symmetric Li metal cell using the ion gel as the electrolyte layer showed stable plating/stripping cycles at a current density of 0.5 mA/cm^2^ over 310 cycles. Furthermore, as a demonstration, [Li | ion gel | LiFePO_4_] full cells were assembled using the ion gel healed after cutting, as well as the unhealed cut ion gel (Figure 8c). Notably, the full cell fabricated with the unhealed cut ion gel quickly faded within 10 cycles, which is possibly ascribable to inhomogeneous Li plating/stripping and consequent Li dendrite formation around the cut area due to its mechanical weakness. In sharp contrast, the full cell fabricated with the healed ion gel exhibited stable cycling for 50 cycles, which indicates that Li dendrite growth was effectively suppressed.

Self-healable ion gels that use solvate ionic liquids (SILs) were recently fabricated for the first time by D’Angelo and Panzer and used as gel electrolytes in lithium secondary batteries [100]. Certain concentrated mixtures of lithium salts and solvents (ligand molecules) are categorized as SILs, in which the ligand molecules strongly coordinate to the lithium cations; therefore, they consist of solvated cations and anions with few free ligand molecules [101–104]. Because of their good lithium-ion-transport properties, SILs are considered to be promising electrolytes for next-generation lithium secondary batteries [105–107]. The self-healable solvate ion gels were synthesized by the in situ polymerization of two zwitterionic monomers, namely 2-methacryloyloxyethyl phosphorylcholine (MPC) and sulfobetaine vinylimidazole (SBVI) in [Li(G4)][NTf_2_], a tetraglyme-based SIL (Figure 8d). The ion gel was formed without chemical crosslinkers due to the noncovalent interactions of the zwitterionic moieties. The mechanical properties of the solvate ion gels were found to be greatly affected by the MPC to SBVI composition ratio. The solvate ion gel became stiff and brittle with increasing SBVI content, which implies that the SBVI units tend to self-associate through dipole-dipole interactions and act as strong crosslinking points. On the other hand, MPC-rich solvate ion gels showed soft and stretchable characteristics. In addition, the lithium ion transference number was significantly higher for the MPC-rich solvate ion gels. These results indicate the existence of attractive interactions between the MPC units and the SIL. The MPC-rich solvate ion gel showed self-healing behavior due to its dynamic crosslinking nature. The cut ion gel was healed by reconnecting the cut surfaces at 50°C for 1 h (Figure 8e). Furthermore, the solvate ion gel was used as an electrolyte layer in a [graphite | electrolyte | NCM(523) (LiNi_0.5_Co_0.2_Mn_0.3_O_2_)] full cell. Although significant capacity fading was observed, the cell with the solvate ion gel performed slightly better than that with the neat SIL (Figure 8f).

## 5. Conclusion

Ion gels are a new class of soft material that offer unique physicochemical properties inherent to ILs; hence, they are promising for use in diverse electrochemical applications. In this article, we overviewed recent progress on functional ion gels that can heal mechanical damage through application of an external stimulus or in an autonomous fashion. Photo-healing is a very attractive strategy due to the non-invasiveness and high spatiotemporal controllability of light. In addition, thermally healable ion gels that take advantage of the high thermal stability, non-volatility, and non-flammability of ILs have been developed. Furthermore, based on supramolecular chemistry and dynamic covalent chemistry, the number of reports of self-healing ion gels that do not require external stimuli have gradually increased. Through the precise design of polymer architectures that exploit interactions between the polymer and the IL, ion gels with self-healing abilities as well as other functionalities, such as high stretchability and toughness, can be created. Despite these successes, whether or not the self-healing functions of ion gels can truly contribute to high durability against mechanical loads under the practical conditions that wearable/stretchable devices are expected to operate in remains unclear. Furthermore, as with other self-healing materials, improving creep behavior is another difficult challenge for self-healing ion gels that are reversibly crosslinked [108]. Insightful understanding and control of the complicated interactions between cations, anions, and polymers is critical for future development and for realizing self-healing ion gels that overcome the hurdles presently faced for practical applications.
